# Perioperative Anesthetic Challenges in the Management of an Early Periprosthetic Femoral Fracture Following Total Knee Arthroplasty in a High-Risk Patient: A Case Report

**DOI:** 10.7759/cureus.107299

**Published:** 2026-04-18

**Authors:** Sandar Shune Let Aung, Zun Win, Thet Su Kyi Win, Phyo Aung Kyaw, Aminath Meesan, Thet H Naing

**Affiliations:** 1 Anesthesiology, Indira Gandhi Memorial Hospital, Male', MDV; 2 Anesthesiology, Kulhudhuffushi Regional Hospital, Kulhudhuffushi City, MDV; 3 Medicine, Indira Gandhi Memorial Hospital, Male', MDV; 4 Emergency Medicine, Indira Gandhi Memorial Hospital, Male', MDV

**Keywords:** anterior femoral notching, combined spinal epidural anesthesia, early postoperative complication, femoral nerve block, obesity, orthopedic anesthesia, perioperative pain management, periprosthetic distal femoral fracture, total knee arthroplasty, ultrasound-guided regional anesthesia

## Abstract

Early periprosthetic distal femoral fractures following total knee arthroplasty (TKA) are uncommon but clinically significant complications requiring urgent surgical intervention. These cases pose substantial anesthetic challenges, particularly in obese patients and during early reoperation. We report the anesthetic management of a 47-year-old female patient (BMI 39 kg/m²) who underwent bilateral TKA followed by surgical fixation of a periprosthetic femoral fracture 25 days later. Both procedures were performed under combined spinal-epidural anesthesia with ultrasound-guided femoral nerve block for analgesia. This case highlights the importance of individualized anesthetic planning, neuraxial techniques, and multimodal analgesia in optimizing perioperative outcomes.

## Introduction

Total knee arthroplasty (TKA) is a widely performed procedure for the management of end-stage knee osteoarthritis and provides significant improvements in pain and functional outcomes. However, complications such as periprosthetic distal femoral fractures, although uncommon (0.3%-2.5%), can occur and are associated with significant morbidity and complex management requirements [1,2].

These fractures often occur in the supracondylar region and may develop due to a combination of patient-related and surgical factors. Patient-related risk factors include advanced age, osteoporosis, and increased body mass index, all of which may compromise bone strength and increase susceptibility to fracture. Obesity, in particular, is associated with increased mechanical stress across the prosthetic joint and presents additional perioperative anesthetic challenges, including airway management difficulties and altered pharmacokinetics [3].

The choice of anesthetic technique plays a crucial role in optimizing outcomes in orthopedic procedures. Regional anesthesia, including neuraxial techniques such as spinal and epidural anesthesia, has been shown to provide effective intraoperative conditions while reducing perioperative complications compared to general anesthesia [4]. Combined spinal-epidural (CSE) anesthesia offers the advantages of rapid onset of anesthesia and the ability to extend analgesia into the postoperative period.

In addition to neuraxial techniques, peripheral nerve blocks have become an integral component of perioperative analgesia in TKA. Ultrasound-guided femoral nerve block (FNB) has been shown to provide superior analgesia, reduce opioid consumption, and facilitate early mobilization [5]. The potential role of combining femoral and sciatic nerve blocks has also been explored, although the additional benefit remains a subject of debate [6].

Despite their analgesic advantages, FNBs may be associated with quadriceps weakness, which can increase the risk of falls and delay mobilization in the postoperative period [7]. Therefore, careful patient selection and monitoring are essential.

Multimodal analgesia strategies that combine neuraxial anesthesia, peripheral nerve blocks, and systemic analgesics are widely recommended to optimize postoperative pain control and enhance recovery following TKA [8].

We present a case highlighting perioperative anesthetic challenges in a patient undergoing early reoperation for a periprosthetic distal femoral fracture following TKA, with particular emphasis on the role of combined neuraxial anesthesia and ultrasound-guided FNB.

This case is unique due to the occurrence of early periprosthetic fracture within 25 days, requiring reoperation in a high-risk patient with obesity, atrial fibrillation, and uncontrolled diabetes, highlighting specific anesthetic challenges and decision-making.

## Case presentation

**Figure 1:**
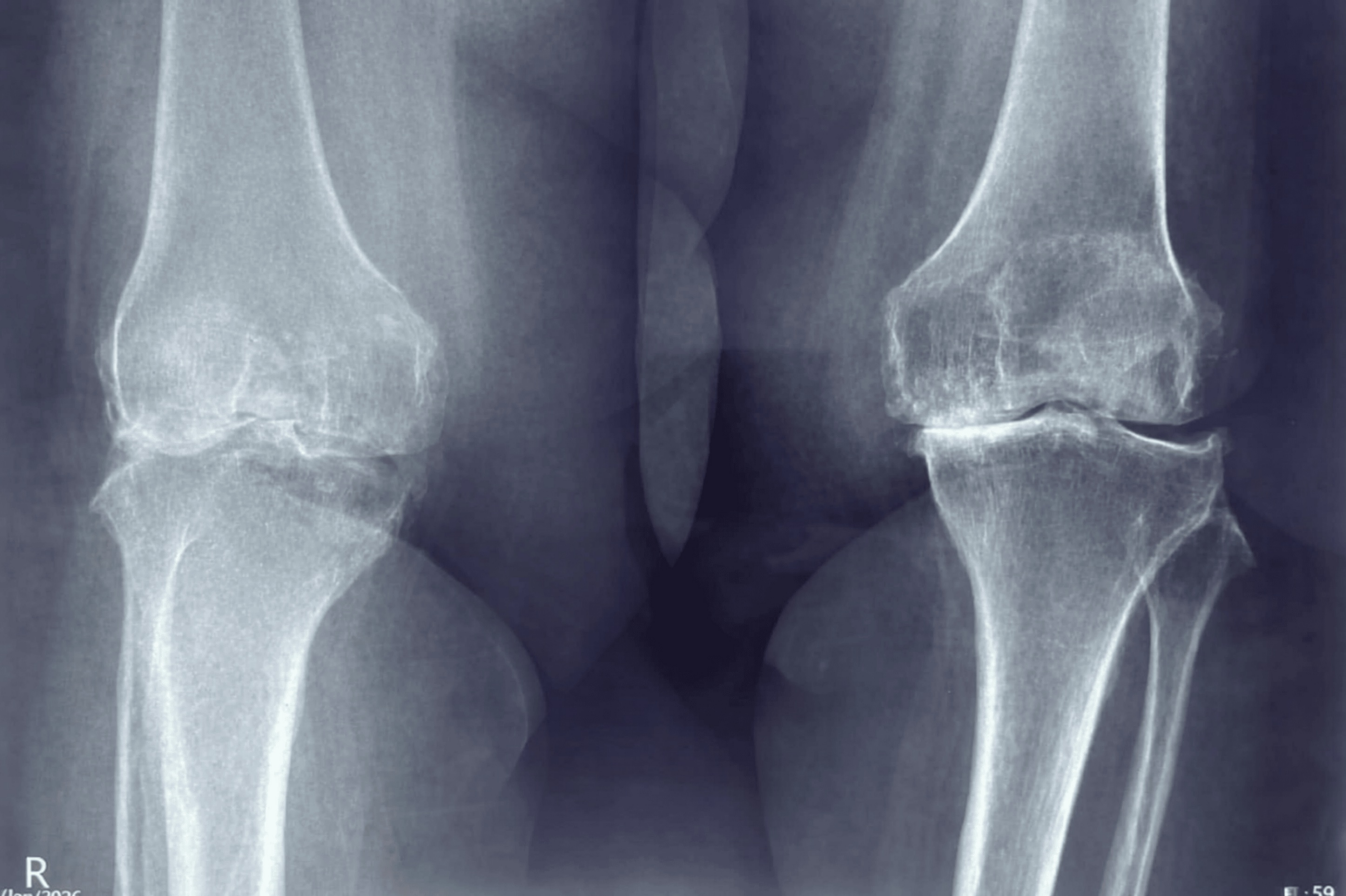
Preoperative radiograph of the knee demonstrating advanced osteoarthritic changes.

A 47-year-old woman with severe bilateral knee osteoarthritis, with persistent severe pain (pain score 8/10) refractory to multiple conservative treatment modalities, was scheduled for elective bilateral TKA. (Figure 1). Her medical history included hypertension, controlled with telmisartan and clonidine, and atrial fibrillation (AF) for which she was not receiving antiarrhythmic therapy. She also had poorly controlled type 2 diabetes mellitus, with a glycated hemoglobin (HbA1c) of 9.8%. The patient had class II obesity (BMI 39 kg/m²) and was classified as American Society of Anesthesiologists (ASA) Physical Status III.

She denied symptoms suggestive of heart failure, including orthopnea, paroxysmal nocturnal dyspnea, or exertional chest pain, and had good functional capacity (MET >4).

Preoperative evaluation

Preoperative laboratory investigations showed normal renal and liver function, normal electrolyte levels, and no evidence of anemia or coagulopathy. Chest radiography demonstrated cardiomegaly without pulmonary congestion or signs of heart failure. Electrocardiography confirmed atrial fibrillation with a controlled ventricular response.

Transthoracic echocardiography revealed preserved left ventricular systolic function with an ejection fraction of approximately 60%, without intracardiac thrombus or significant valvular pathology.

Internal medicine consultation was obtained for perioperative optimization. The patient was initiated on a basal-bolus insulin regimen, and variable rate intravenous insulin infusion (VRIII) was planned to maintain intraoperative blood glucose levels within a target range of 140-180 mg/dL. LMWH thromboprophylaxis (enoxaparin 40 mg subcutaneously once daily) was initiated on admission, withheld 12 hours prior to surgery, and restarted 12 hours after epidural catheterization. Epidural catheter removal was performed after ensuring a 12-hour interval since the last LMWH dose, and the subsequent dose was administered four hours after catheter removal.

Airway assessment suggested a potentially difficult airway (Mallampati III and increased neck circumference), although mouth opening and neck mobility were adequate. Advanced airway equipment, including a video laryngoscope and supraglottic devices, was prepared.

Primary surgical procedure

For the primary bilateral TKA, CSE anesthesia was performed. Standard monitoring included electrocardiography, non-invasive blood pressure, and pulse oximetry. Intravenous access was secured, and baseline vitals were recorded.

With the patient in the sitting position, the epidural space was identified at the L3-L4 interspace, and an epidural catheter was inserted with single attempt. Spinal anesthesia was administered with 0.5% hyperbaric bupivacaine 15 mg and fentanyl 25 µg, achieving a sensory blockade up to T10.

**Figure 2:**
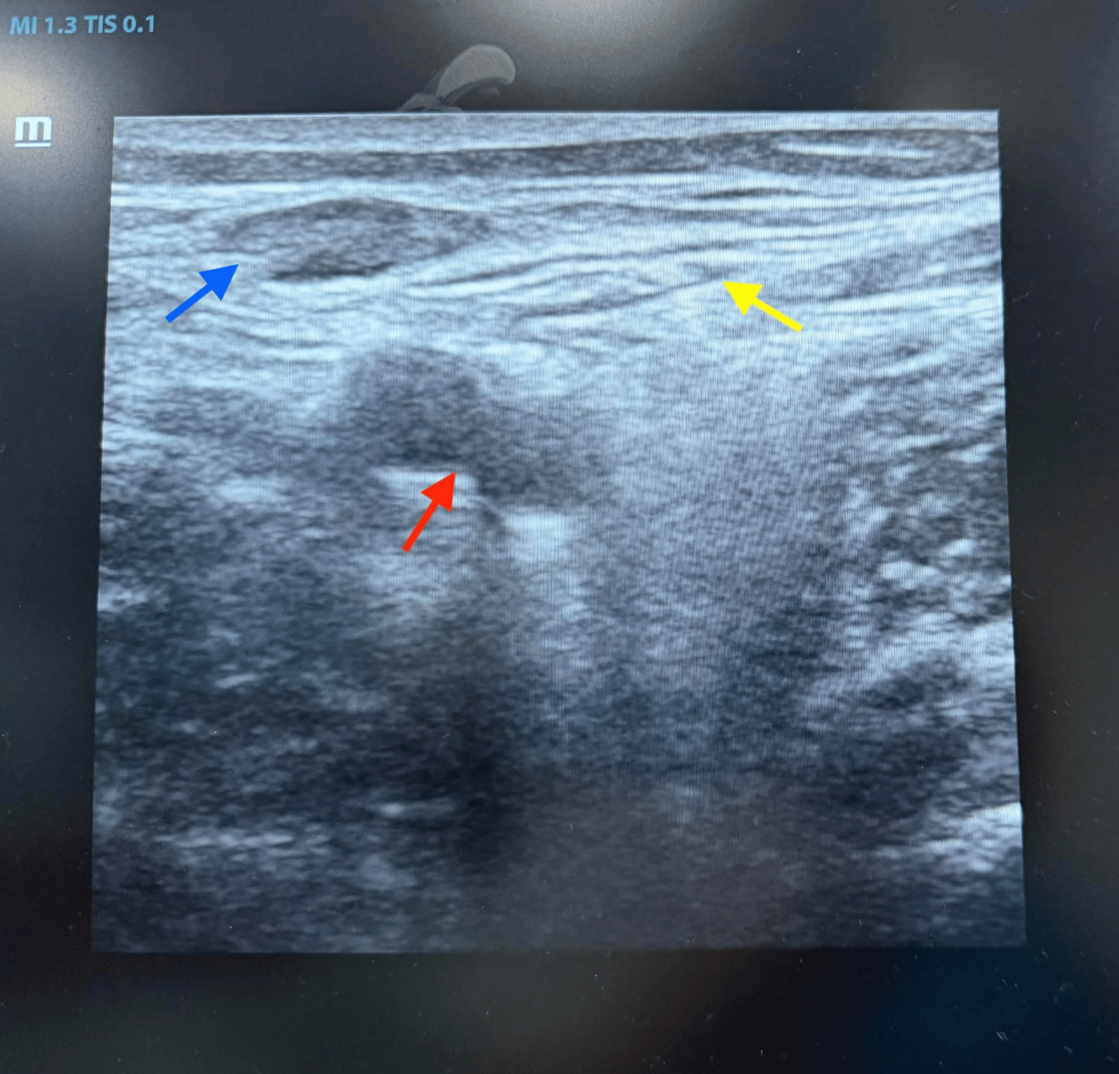
Ultrasound image of the femoral neurovascular bundle. Ultrasound image demonstrating the femoral neurovascular structures. The femoral artery (red arrow) is seen as a pulsatile anechoic structure, the femoral vein (blue arrow) lies medial to the artery, and the femoral nerve (yellow arrow) appears as a hyperechoic structure lateral to the artery.

Epidural supplementation with 0.25% bupivacaine was used to maintain anesthesia during the prolonged procedure. For postoperative analgesia, ultrasound-guided bilateral adductor canal blocks were performed using 15 mL of 0.25% bupivacaine per side, along with IPACK blocks using 15 mL of 0.25% bupivacaine per side. The total local anesthetic dose was maintained within safe limits, and the patient was monitored for signs of local anesthetic systemic toxicity (Figure 2).

The procedure was prolonged due to surgical complexity; however, the patient remained hemodynamically stable with no signs of LAST. Blood glucose was maintained within the target range using VRIII.

Postoperative course after primary surgery

Postoperative analgesia was maintained with epidural infusion of 0.125% bupivacaine with fentanyl (2 µg/mL), supplemented with intravenous acetaminophen and non-steroidal anti-inflammatory drugs as appropriate. The initial postoperative course was uneventful.

Development of periprosthetic fracture

**Figure 3:**
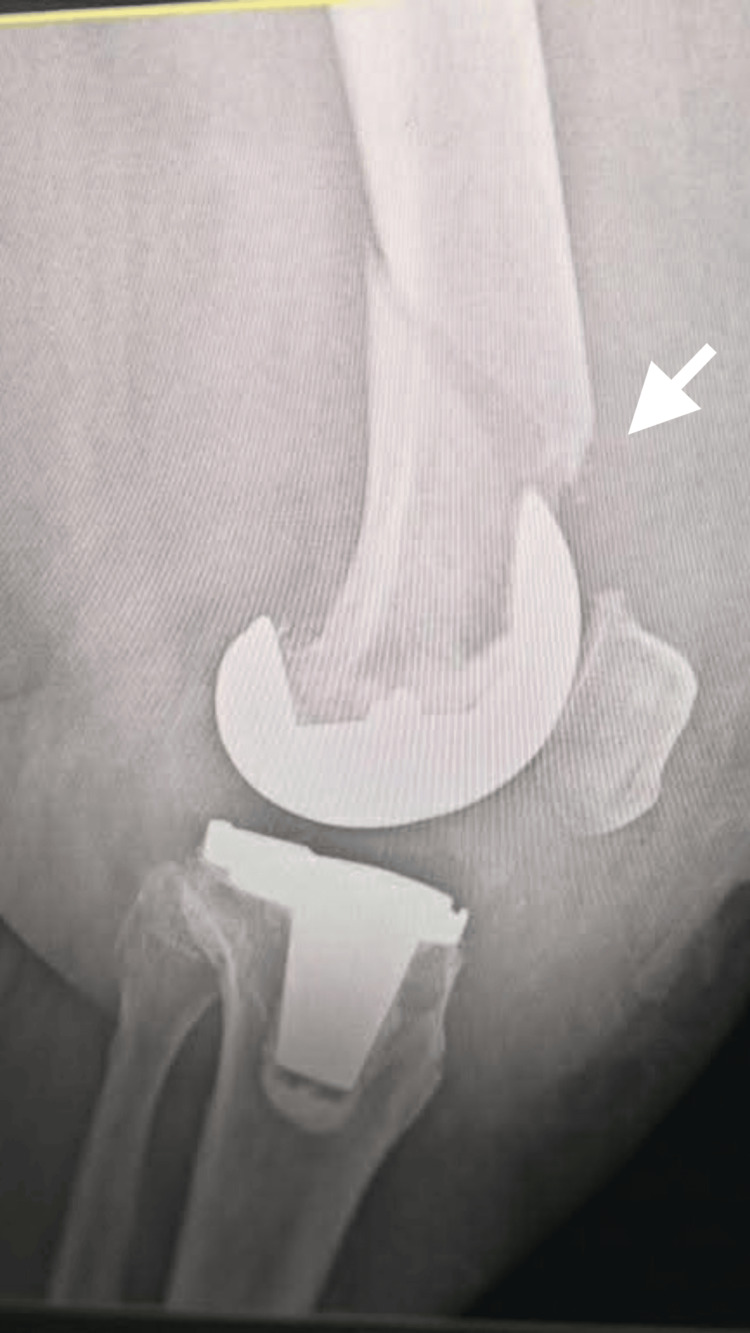
Radiograph demonstrating a supracondylar periprosthetic distal femoral fracture above the femoral component.

On postoperative day 25, the patient developed severe left knee pain with inability to bear weight. Examination revealed localized tenderness, swelling, and restricted movement. A radiograph confirmed a supracondylar periprosthetic distal femoral fracture above the femoral component (Figure 3).

Second surgical procedure

The patient underwent open reduction and internal fixation with a distal femoral locking plate. Anesthetic management was considered high risk due to early reoperation and multiple comorbidities. Combined spinal-epidural anesthesia was again selected to minimize systemic complications and avoid airway manipulation.

Spinal anesthesia was administered with 0.5% hyperbaric bupivacaine 12 mg and fentanyl 25 µg, achieving adequate sensory blockade. An ultrasound-guided FNB was performed on the affected side using 20 mL of 0.25% bupivacaine to facilitate positioning and perioperative analgesia.

Standard intraoperative monitoring, including ECG, blood pressure, oxygen saturation, and blood glucose, was maintained. Glycemic control was achieved using VRIII, and the patient remained hemodynamically stable.

**Figure 4:**
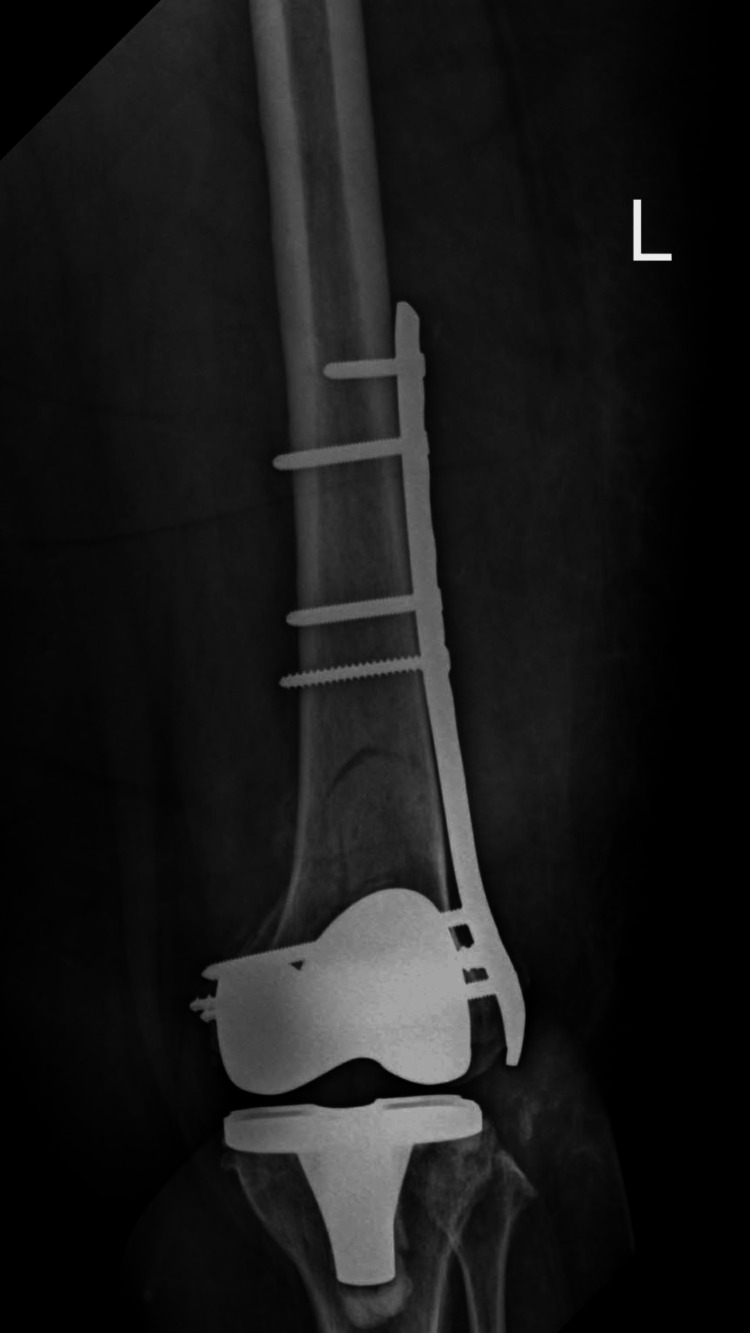
Postoperative radiograph demonstrating fixation of the periprosthetic distal femoral fracture with a distal femoral locking plate.

Surgical fixation was completed successfully, and a postoperative radiograph confirmed satisfactory alignment (Figure 4).

Postoperative recovery

Postoperative analgesia followed a multimodal approach using epidural infusion (0.125% bupivacaine with fentanyl 2 µg/mL), intravenous acetaminophen, and opioid rescue as required.

The thromboprophylaxis protocol and management of epidural catheter insertion and removal were the same as in the primary surgery. The patient demonstrated good pain control, stable cardiovascular status, and gradual improvement in mobility during rehabilitation.

## Discussion

Periprosthetic distal femoral fractures are uncommon but clinically significant complications following TKA, with a reported incidence ranging from 0.3% to 2.5% in primary procedures [1,2]. These fractures often require surgical fixation and present substantial perioperative challenges, particularly when they occur early after the index surgery. In the present case, fracture occurrence within 25 days highlights the vulnerability of the distal femur during the early postoperative period, especially in patients with additional risk factors such as obesity. Early reoperation within 30 days further increases anesthetic complexity due to incomplete physiological recovery from the initial procedure.

Patient-related comorbidities played a crucial role in anesthetic planning. Obesity is associated with increased mechanical loading across the prosthetic joint and distal femur, as well as increased perioperative anesthetic risk due to altered respiratory mechanics, airway challenges, and changes in drug pharmacokinetics [3]. Additionally, AF, even in the absence of active pharmacological management, increases the risk of perioperative hemodynamic instability and thromboembolic complications. Careful cardiovascular assessment, including preserved ejection fraction and absence of intracardiac thrombus on echocardiography, supported the anesthetic plan. These considerations are consistent with previous reports highlighting increased perioperative risk in patients with cardiovascular comorbidities and poorly controlled diabetes [3,4]. Although formal cardiac risk scoring was not performed, the patient’s good functional capacity (MET >4) further supported proceeding with neuraxial anesthesia.

Uncontrolled diabetes mellitus (HbA1c 9.8%) further increased perioperative risk due to its association with infection, impaired wound healing, and cardiovascular instability. Intraoperative glycemic control using VRIII allowed maintenance of blood glucose within a target range and minimized metabolic fluctuations, which is essential in high-risk surgical patients.

The choice of anesthetic technique was particularly important. Regional anesthesia, including neuraxial techniques such as CSE, has been shown to improve perioperative outcomes in orthopedic surgery by reducing thromboembolic complications, improving analgesia, and minimizing systemic adverse effects [4]. In this case, CSE provided reliable anesthesia for both the primary procedure and the reoperation while allowing flexibility for prolonged surgery and postoperative pain management.

The regional analgesic approach differed between the two procedures. During the primary surgery, an adductor canal block combined with an IPACK block was used to preserve quadriceps strength and facilitate early mobilization. However, during the second procedure, FNB was selected. Previous studies have demonstrated that FNB provides superior analgesia, reduces opioid consumption, and facilitates early mobilization following TKA [5]. Although FNB is associated with quadriceps weakness, it offers a more proximal and denser sensory blockade compared to adductor canal techniques. In this case, severe pain due to an acute fracture necessitated a more robust analgesic approach to facilitate patient positioning for neuraxial anesthesia and ensure procedural safety.

The potential role of additional nerve blocks, such as a sciatic nerve block, has also been described. While combining femoral and sciatic nerve blocks may provide more comprehensive analgesia, the incremental benefit must be balanced against increased procedural complexity and potential risks [6]. In this patient, FNB alone provided adequate analgesia without the need for additional blocks.

Despite its analgesic benefits, FNB may result in quadriceps muscle weakness, which can impair mobilization and increase the risk of falls [7]. Therefore, careful postoperative monitoring and structured rehabilitation are essential, particularly in patients undergoing fracture fixation.

Multimodal analgesia, combining neuraxial techniques, peripheral nerve blocks, and systemic analgesics, remains the cornerstone of postoperative pain management in TKA [8]. In this case, analgesic strategies were selectively tailored to balance effective pain control with early mobilization rather than routine use of all modalities; the combination of epidural infusion, FNB, and systemic analgesics provided effective pain relief and facilitated early rehabilitation.

The second surgical intervention posed additional anesthetic challenges due to early reoperation, acute pain, and multiple comorbidities including AF and uncontrolled diabetes. A carefully titrated neuraxial technique, combined with ultrasound-guided regional anesthesia and vigilant perioperative monitoring, allowed safe and effective anesthetic management.

Overall, this case highlights the importance of individualized anesthetic planning in complex orthopedic patients. The integration of neuraxial anesthesia, ultrasound-guided nerve blocks, and multimodal analgesia can optimize perioperative outcomes, particularly in high-risk early reoperative scenarios.

## Conclusions

Early periprosthetic distal femoral fractures following TKA present significant perioperative anesthetic challenges, particularly in patients with multiple comorbidities undergoing early reoperation. This case demonstrates that a tailored anesthetic approach using combined spinal-epidural anesthesia with ultrasound-guided regional nerve blocks can provide effective intraoperative conditions and optimize postoperative analgesia.

Careful dose adjustment of neuraxial anesthesia, appropriate selection of regional techniques, and implementation of multimodal analgesia are essential to maintain hemodynamic stability, facilitate safe patient positioning, and support early rehabilitation. Multidisciplinary coordination is also critical in optimizing perioperative outcomes. This case underscores the importance of individualized anesthetic strategies in early reoperative settings, where comorbidities and surgical timing significantly influence perioperative risk. Further clinical studies are warranted to better define optimal anesthetic approaches in such high-risk scenarios.
